# Characterization of a panARS-based episomal vector in the methylotrophic yeast *Pichia pastoris* for recombinant protein production and synthetic biology applications

**DOI:** 10.1186/s12934-016-0540-5

**Published:** 2016-08-11

**Authors:** Andrea Camattari, Amelia Goh, Lian Yee Yip, Andy Hee Meng Tan, Sze Wai Ng, Anthony Tran, Gaowen Liu, Ivan Liachko, Maitreya J. Dunham, Giulia Rancati

**Affiliations:** 1 Bioprocessing Technology Institute (A-STAR), 20 Biopolis Way, #06-01 Centros, Singapore, 138668 Singapore; 2 Institute of Medical Biology (A-STAR), 8a Biomedical Grove, #06-06, Singapore, 138648 Singapore; 3 Department of Genome Sciences, University of Washington, Seattle, WA USA

**Keywords:** *P. pastoris*, panARS, BFP, Episomal plasmid, Interclonal variability, Digital droplet PCR, In vivo recombination, Synthetic biology

## Abstract

**Background:**

Recombinant protein production in the methylotrophic yeast *Pichia pastoris* largely relies on integrative vectors. Although the stability of integrated expression cassettes is well appreciated for most applications, the availability of reliable episomal vectors for this host would represent a useful tool to expedite cloning and high-throughput screening, ameliorating also the relatively high clonal variability reported in transformants from integrative vectors caused by off-target integration in the *P. pastoris* genome. Recently, heterologous and endogenous autonomously replicating sequences (ARS) were identified in *P. pastoris* by genome mining, opening the possibility of expanding the available toolbox to include efficient episomal plasmids. The aim of this technical report is to validate a 452-bp sequence (“panARS”) in context of *P. pastoris* expression vectors, and to compare their performance to classical integrative plasmids. Moreover, we aimed to test if such episomal vectors would be suitable to sustain in vivo recombination, using fragments for transformation, directly in *P. pastoris* cells.

**Results:**

A panARS-based episomal vector was evaluated using blue fluorescent protein (BFP) as a reporter gene. Normalized fluorescence from colonies carrying panARS-BFP outperformed the level of signal obtained from integrative controls by several-fold, whereas endogenous sequences, identified from the *P. pastoris* genome, were not as efficient in terms of protein production. At the single cell level, panARS-BFP clones showed lower interclonal variability but higher intraclonal variation compared to their integrative counterparts, supporting the idea that heterologous protein production could benefit from episomal plasmids. Finally, efficiency of 2-fragment and 3-fragment in vivo recombination was tested using varying lengths of overlapping regions and molar ratios between fragments. Upon optimization, minimal background was obtained for in vivo assembled vectors, suggesting this could be a quick and efficient method to generate of episomal plasmids of interest.

**Conclusions:**

An expression vector based on the panARS sequence was shown to outperform its integrative counterparts in terms of protein productivity and interclonal variability, facilitating recombinant protein expression and screening. Using optimized fragment lengths and ratios, it was possible to perform reliable in vivo recombination of fragments in *P. pastoris*. Taken together, these results support the applicability of panARS episomal vectors for synthetic biology approaches.

## Background

The methylotrophic yeast *Pichia pastoris* is widely considered an industrial workhorse for recombinant protein production (RPP, [1–3]); insights into *P. pastoris* genomic arrangement [4, 5] and metabolism [6, 7] are starting to accumulate in the literature, as a testament to the interest in this host. Despite an increasing repertoire of molecular tools to enable efficient protein production, including newly identified natural promoters [8], engineered sequences [9, 10], and secretion signals [11], the vast majority of vectors available for RPP are based on genomic integration of expression cassettes in the *P. pastoris* genome. Although in general stable clones derived from genomic integration are preferred for RPP, disadvantages of their use are related to relatively low efficiency of transformation (recently at least mitigated by technical developments [12]), to a certain degree of heterogeneity in protein production due to non-specific integration and to genetic instability of multi-copy integration in presence of stress conditions [13]. The classical solution to this problem—episomal expression vectors—is deemed to alleviate only the chromosomal instability of multi-copy integrative clones, since other reasons of heterogeneity, recently addressed analysing micro engraved *P. pastoris* secretive clones, are hypothesized to be related to stochastic post-translational events, especially relevant in secreted protein expression [14, 15]. Nonetheless, episomal expression systems present advantages such as simpler protocols and higher efficiencies of transformation; however, such tools are unavailable in most non-canonical protein production hosts, often due to lack of efficient replication origins that promote in vivo plasmid replication and maintenance. Recent high-throughput work has identified novel autonomously replicating sequences (ARSs) in different organisms, to bring to light novel regions conferring self-replicating properties and understand their features [16–18]. These elements may help expedite RPP efforts through addition of stable expression plasmids to the available molecular toolkit; moreover, another intriguing possibility is represented by the possibility of self-assembly recombinant DNA fragments in its nucleus, eliminating the cloning process for plasmid assembly [19]; such strategy is theoretically facilitated by self-replication of the assembled fragment in the recipient cells, and has been successfully applied to *Saccharomyces cerevisiae* [20, 21]. In vivo recombination in *P. pastoris* was first observed when a library of *Rhizopus chinensis* lipase mutants was assembled directly by the host and generated a linear expression cassette integrated at the targeted genomic locus. Overlapping ends as short as 50 nucleotides were reported to be sufficient to promote assembly at a relatively high efficiency [22].

In this technical report, we aimed to functionally characterize the use of panARS, a 452-nt element originally isolated from *Kluyveromyces lactis* and synthetically optimized, as well as two endogenous sequences derived from *P. pastoris*, using blue fluorescent protein (BFP) as a reporter protein. Moreover, we evaluated in vivo recombination of a panARS-based vector, to establish its technical feasibility for efficient gene assembly in *P. pastoris*.

## Results and discussion

### Evaluation of ARSs in *Pichia pastoris* GS115

Recently, endogenous autonomously replicating sequences from *P. pastoris* have been identified and described [23]. In order to evaluate the general performance of two sequences in comparison with a wide-range ARS (panARS, [16]), sequences A76 and C937, previously described to possess respectively strong and moderately weak self-replicating activity, were tested in the same genetic context as panARS. To do so, blue fluorescent protein (BFP)-expressing plasmids, containing AOX1 promoter, zeocin resistance cassette and each of the three different ARS sequences, were constructed starting from the commercial vector pSEC-SUMO (see ‘‘Methods’’ section). BFP was selected as reporter gene, due to its fast maturation, high photostability and pH-stability [24–26].

**Fig. 1:**
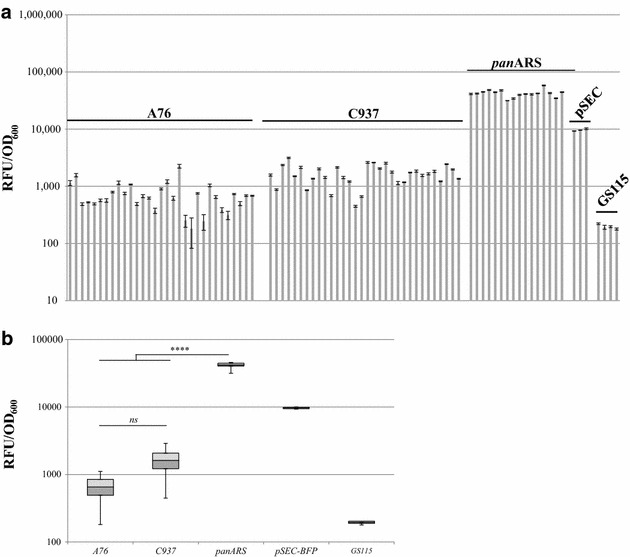
Evaluation of normalized fluorescence of *pan*ARS-, A76- or C937-based plasmids. **a** Histogram and **b** representation of normalized fluorescence level (RFU/OD_600_) of individual clones transformed with A76-, C937-, panARS-based episomal vectors, compared to integrative clones (pSEC) and parental strain (GS115) (*ns* Sidak’s multiple comparison test on one-way ANOVA, *α* 0.05; *one-way ANOVA, p < 0.05)

Transforming *P. pastoris* GS115 with a circular plasmid carrying either A76, C937 or panARS sequences, a substantial number of colonies was obtained. At least 16 independent colonies carrying A76-, C937- or panARS-based BFP-expressing plasmids were tested for fluorescence emission at 452 nm after 48 h of cultivation using sorbitol and methanol as carbon sources (Fig. 1a). Co-feeding with sorbitol and methanol allowed shortened cultivation time, as sorbitol is in general more easily assimilated than methanol, without repressing the *AOX1* promoter [27]. Fluorescence emission was then normalized on OD values as an estimation of biomass. Interestingly, the BFP expression level for A76 or C937-carrying strains was significantly lower than either panARS-based or integrative vector strains (Fig. 1a, b). As shown in Fig. 1a, three independent colonies of *P. pastoris* GS115, carrying an integrative version of the same plasmid (pSEC) and selected from the middle of the expression landscape (data not shown), were used as positive controls and as benchmarks for episomal vectors evaluation (Fig. 1a). Such average clones, typically carrying a single copy of expression cassette genomically integrated, were outperformed by panARS-based clones, while showing higher and more consistent BFP expression level than either A76- or C937-based vectors (Fig. 1a). In an attempt to determine the reason of such performance, the average copy number of the different constructs was determined using digital droplet PCR. Two primer sets were designed either on the zeocin—pEM72 promoter fragment or within the BFP coding sequence. Three representative transformants per construct (integrative, panARS-, A76- and C937-based vectors) were tested (Table 1). Clones D4 and E2 (which normalized BFP fluorescence showed a nearly identical level) approximately integrated one copy per genome, while clone G5, despite a very similar fluorescence level, presented approximately two copies of BFP-expressing vector per genome. A possible explanation for this observation is that the extra integrated copy might have been integrated in a poorly transcriptionally active genome region: although attempts to identify such transcriptionally inactive region were carried out, it was not possible to clearly identify the location of the second integrated copy, leaving the possibility of low transcription rate for the second copy a speculation at the moment. PanARS-based vector transformants showed significant variation in copy number between different clones, despite a very similar normalized fluorescence level, suggesting the involvement of post-translational (or epigenetic) factors in protein expression for *P. pastoris*, consistently with the recent literature [14], as previously mentioned; further investigations will be required to fully clarify the bottlenecks involved. Finally, clones carrying either A76- or C937-based vectors showed copy number lower than one, strongly indicating an asymmetric distribution of plasmids within the population; for this reason, and since the average expression level of plasmids carrying A76 or C937 was significantly lower than panARS-based vectors, only the latter was selected for further evaluation and development.

**Table 1:** Copy number determination for integrated or episomal BFP-expressing vectors

Construct/clone	Copy number Zeo Set 1	Copy number BFP Set 1
Integrative/D4	0.87	1.06
Integrative/G5	1.91	2.20
Integrative/E2	0.98	1.20
panARS/B8	18.26	19.08
panARS/E8	13.00	14.14
panARS/C10	6.11	6.48
A76/D1	0.42	0.39
A76/F2	0.66	0.66
A76/F3	0.45	0.41
C937/D3	0.89	0.88
C937/C1	0.67	0.59
C937/A6	0.74	0.78

### Evaluation of potential positional effect of panARS

**Fig. 2:**
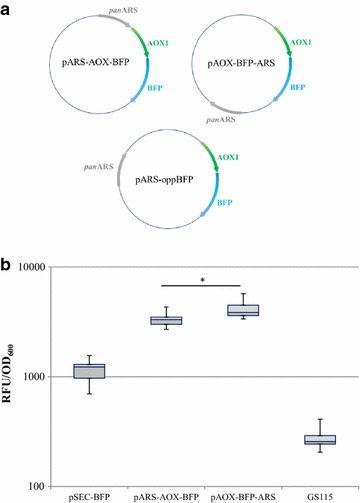
Evaluation of positional effects for panARS. **a** Schematic representation of constructs generated to evaluate putative positional effects for the panARS sequence. **b** Box-plot representation of normalized fluorescence emission (RFU/OD_600_) of *P. pastoris* independent colonies carrying pARS-AOX-BFP or pAOX-BFP-ARS (*, unpaired t-test, p < 0.05)

Autonomously replicating sequences can influence RNA transcription machinery in addition to their role in DNA replication [28, 29]: we therefore evaluated the positional effect of panARS on protein expression. PanARS was placed either upstream of the methanol-inducible *AOX1* promoter, downstream of the *AOX1* terminator (pAOX-BFP-ARS) or downstream of the cytochrome c terminator, on the opposite side of the BFP coding sequence (pARS-oppBFP) (Fig. 2a). When panARS was placed opposite of BFP or upstream of the *AOX1* promoter, the same BFP expression level was detected (data not shown); however, a slight beneficial positional effect was observed when origins of replication were placed downstream terminator sequences in episomal plasmids (Fig. 2b), (unpaired *t* test, p < 0.05). As the substantial equivalence of expression levels detected with pARS-AOX-BFP and pARS-oppBFP suggests, further analysis will be required to properly clarify whether the vicinity of panARS to the *AOX1* terminator is responsible for the observed slight performance improvement, possibly reinforcing transcription termination of mRNA.

### Specific fluorescence evaluation at the single cell level

**Fig. 3:**
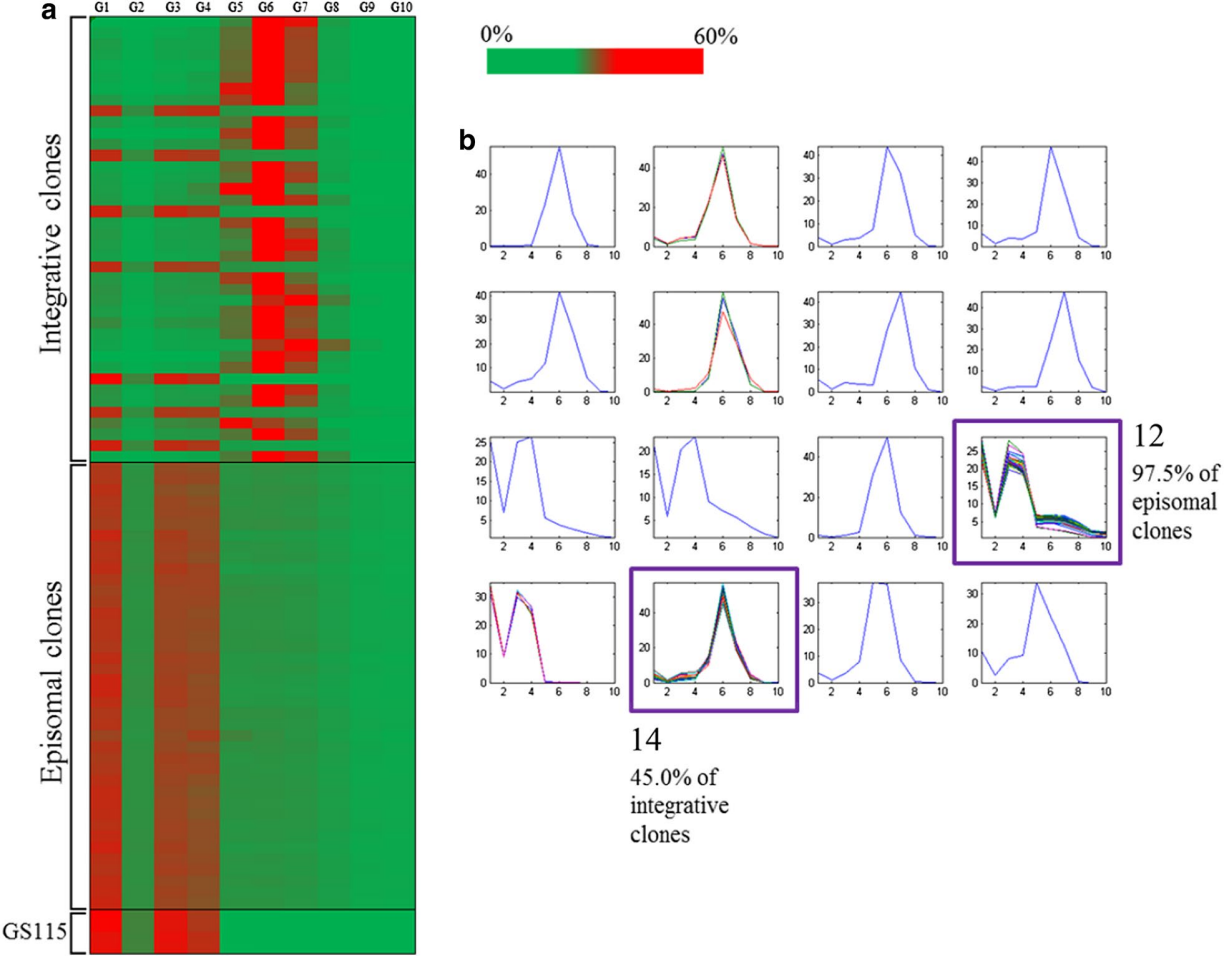
Flow cytometry analysis and hierarchial clustering of individual clones carrying episomal or integrative BFP-expressing vectors. **a** The overall BFP fluorescence signal was distributed between 10 equally spaced gates as percentage of fluorescence (being the total fluorescence for every clone equal to 100 %).* Color-coded* gating for frequencies of BFP fluorescence events in cultures derived from 40 individual clones expressing either integrative or episomal vectors (*Green* 0 %, *Red* 60 %). **b** Hierarchial clustering of fluorescence distribution; cluster 12 (containing 97.5 % of episomal clones, and 14, containing 45 % of integrative clones, are highlighted—see text and ‘‘Methods’’ section for details)

To evaluate the intra population variation in the BFP expression from either episomal on integrative plasmids, samples from deep-well plate cultivation of integrative or episomal BFP producers were analysed by flow cytometry. 40 individual colonies, transformed with either integrative or episomal BFP expression plasmids, were cultivated in 96-well format and induced for BFP expression. After 48 h of cultivation, colonies were tested for specific fluorescence emission (normalized on OD_600_ as an estimation of biomass) and analysed via flow cytometry. The fluorescence channel corresponding to BFP emission at 452 nm was gated applying an equidistant threshold, providing the percentage of cells populating 10 different gates (named from G1 to G10), from zero (G1) to highest (G10) BFP emission. The percentage of fluorescent cells populating the different gates is depicted in a heat map (Fig. 3a). When hierarchial clustering was applied, 97.5 % of episomal clones were grouped in the same cluster (cluster 12, Fig. 3b), whilst integrative clones were more heterogeneous, strongly suggesting increased variation across the population of several independent clones (approximately 45 % of integrative clones were clustered in cluster 14, Fig. 3b). The increase in BFP production at the macroscopic level could have resulted from two different events at the microscopic level: (a) every single cell in the population could present a moderate increase in BFP emission, resulting in an increased median value for emission, or (b) a sub-population of high producers, driven by asymmetric plasmid distribution within the growing population. Analysing flow cytometry profiles, it appears that episomal expression of BFP in the whole population is higher, when compared to integrative expression, due to a polarization of the cell population, resulting in a higher density of both low- and high-producers. Such an observation was confirmed by analysing single clones, cultivated in deep-well plates or shake flasks, where the percentage of fluorescent events corresponding to high BFP emission was significantly higher for clones carrying episomal vectors (data not shown). Among the reasons of such instability there is plasmid stability, even in presence of selective pressure. Preliminary results on a subset of episomal clones showed that plasmid stability was 97.3 % ± 3.80 and 39.04 ± 5.54 (expressed as percentage of cells retaining the plasmid) when growth was performed for 10.8 generations in YPD media supplemented or not with the selective marker zeocin, respectively. Since cultivation before BFP assessment took place in presence of zeocin after propagation of <10 generations, this suggests that such propagation rates for panARS vectors appear to exclude plasmid instability as the reason for the relatively large number of non-fluorescent single cells measured, since cultivation took place in presence of zeocin and for lower generation numbers. Further study will be required to analyse such effects over longer generation times, and how the selection marker might influence this outcome, since it must be noted that copy number variation was noted taken into account when plasmid stability was determined.

### In vivo recombination: proof-of-principle

**Fig. 4:**
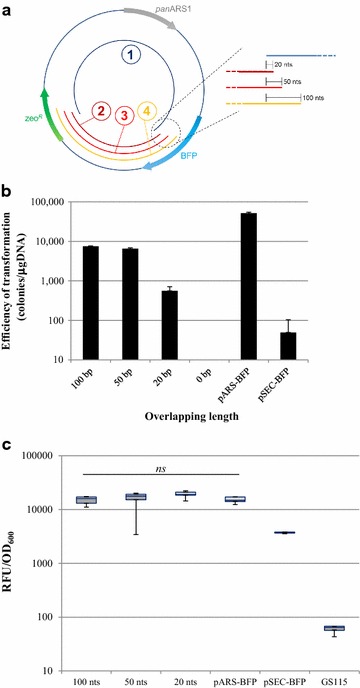
Proof-of-principle of in vivo recombination (two-fragment assembly). **a** Schematic diagram of fragments used for testing in vivo recombination using pARS-BFP as template. **b** Frequencies of transformation obtained using overlaps of 100, 50, or 20 nucleotides between fragment 1 and fragments 2, 3, or 4. **c** BFP fluorescence signal obtained from 8 independent colonies randomly selected from the different pools (*ns* non-significant, according to Sidak’s multiple comparison test on one-way ANOVA, *α* 0.05)

One of the goals of developing an efficient episomal vector for *P. pastoris* was the possible application of in vivo recombination (self-assembly of an expression cassette or plasmid directly by the cell machinery) in *P. pastoris*, as such techniques would allow faster, cheaper, and higher throughput screening of bioparts from this organism for synthetic biology, as in *S. cerevisiae* [30, 31]. In order to test whether panARS-based expression would be a suitable tool for in vivo recombination in *Pichia pastoris*, the following strategy was conceived (Fig. 4a). As a proof-of-principle, 4 fragments were PCR-amplified using panARS-BFP as a template and gel-purified. To minimize the integration of the fragment carrying the zeocin marker, which would have resulted in a background of non-fluorescent colonies (given the tendency of *P. pastoris* to integrate linear DNA fragments in its genome), the coding sequence for the selection marker was split between fragment 1 and either fragments 2, 3 or 4. These fragments were designed to overlap each other by 20, 50, or 100 nucleotides over a backbone fragment (fragment 1; Fig. 4a). Theoretically, only an in vivo reconstituted plasmid could provide an intact selection marker, (zeocin resistant, non-fluorescent colonies), whilst the individual fragments (1 plus either 2, 3 or 4), even if integrating, would not be able to do so. Transformation efficiency, which in this case implies that DNA must be not only incorporated, but also assembled by the cell machinery to yield a growing colony, was measured. When single fragments were used to transform *P. pastoris* GS115, no colonies were obtained (data not shown), whereas when two fragments were co-transformed a significant efficiency of transformation was detected (Fig. 4b). Such efficiency dropped when the overlapping region was trimmed to 20 nucleotides: the total number of colonies obtained in this case was an average of 152.4 ± 41.7, compared with approximately a number ten times higher when the overlapping regions were of 100 and 50 nucleotides in length. Although the efficiency of transformation was lower than in other cases, the 20-nt overlap strategy provides a transformation/ligation efficiency still compatible with recombinant protein production or colony screening, with the advantage that 20-nucleotide overlaps can more cheaply and easily be introduced into PCR primers than 100- or 50-nucleotide overlaps. In addition, as shown in Fig. 3b, the macroscopic interclonal variability of transformants with the panARS vector is limited, minimizing the required screening throughput for productive clones. Eight independent colonies, obtained from various transformation events with different fragments, were tested for BFP production (Fig. 4c), confirming that the resulting clones were expressing BFP at a statistically identical level in respect to the control strains transformed using the full vector pARS-BFP (one-way ANOVA test, p < 0.05, and Sidak’s multiple comparison test on one-way ANOVA). Genomic DNA was extracted from three independent clones from the different sets and used to transform *E. coli*. Sequencing of plasmid DNA extracted from bacterial colonies confirmed the identity of the reconstituted plasmid. No *E. coli* colonies were obtained when genomic DNA from integrated pSEC-BFP clones was used.

### In vivo recombination: cloning simulation

**Fig. 5:**
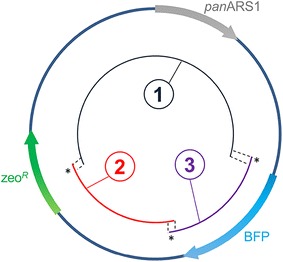
Proof-of-principle of in vivo recombination (three-fragment assembly). Schematic diagram of fragments used for testing in vivo recombination using pARS-BFP as template

**Fig. 6:**
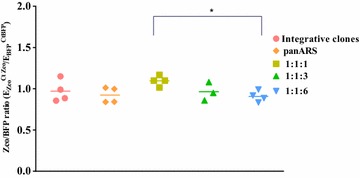
Fragment ratio determination for integrative, episomal and in vivo recombined plasmids (three fragments assembly). Cluster plot for Zeo/BFP fragment ratio for 4 independent clones carrying integrative (*red dot*), episomal (*orange diamond*) or three-fragments in vivo recombined (1:1:1 *lime square*; 1:1:3 *green triangle*—3 independent clones; 1:1:6 *blue inverted triangle*). All distributions were evaluated using one-factor ANOVA analysis (*α* 0.05); the average distribution between sample 1:1:1 and 1:1:6 (*bracketed and indicated by a star*) resulted statistically significant by ANOVA test

The successful proof-of-principle of two-fragment in vivo recombination in *P. pastoris* (Fig. 4) was based on a pre-assembled expression vector. In order to verify whether cloning could be performed adopting such a strategy, a second in vivo recombination experiment was conceived (Fig. 5). A three-fragment strategy was implemented, where two fragments (1 and 2) were designed to reconstitute the backbone vector, and a third one (number 3) encoded the gene of interest (in this instance, BFP). The junctions between the three fragments, marked with a star symbol in Fig. 5, consisted of either 50-nt or 20-nt overlapping regions between the various fragments. Since genome integration of a reconstituted selection marker cassette resulting from recombined fragments 1 and 2 could potentially provide significant background, different molar ratios (from 1:1:1 to 1:1:3 to 1:1:6, respectively for fragments 1, 2 and 3) were tested, for both 20 and 50 nucleotide overlapping regions. Clones were considered positive if their BFP fluorescence was at least higher than 80 % of the difference between negative and positive control (see ‘‘Methods’’ section). Increasing the molar ratio between the backbone fragments and the “gene-of-interest” (BFP) fragment from 1:1:1 to 1:1:3 increased the relative amount of positive clones to >85 % over 72 independent clones tested (Table 2). When the molar ratio was further increased to 1:1:6, ~90 % of the colonies were positive for BFP expression. Taken together, these results suggest that although some background is observed, this method allows fast cloning-independent synthetic biology approaches in *P. pastoris*. Interestingly, when comparing 50-nts overlaps versus 20-nts overlaps, no apparent improvement in performance was observed. To further validate the method, an analysis on the relative abundance of the different fragments was undertaken. Total DNA extracted from randomly-picked clones carrying BFP-expressing vector as integrative, episomal or in vivo recombined constructs was tested for an “assembly ratio” (expressed as the ratio of C_t_ numbers for Zeo and BFP amplicons, normalized by the relative efficiency of the respective primers). In almost all cases, the assembly ratio obtained was close to 1 (Fig. 6); a single clone, characterized by low fluorescent emission failed to yield any plasmid despite repeated efforts, suggesting a relation between plasmid presence and fluorescence level. The fluctuation obtained around a value of assembly ratio of ~1.0) was not statistically different when a fragment ratio of 1:1:3 ratio was compared against 1:1:6; interestingly, a 1:1:1 fragment ratio yielded a statistically significant increase over 1.0 in the assembly ratio (One way ANOVA test, α: 0.05), suggesting a tendency of the zeocin fragment to be present in slightly higher numbers compared to BFP fragment, in accordance with the fact that the theoretical relative abundance of zeocin fragments versus BFP fragment is in fact higher in a 1:1:1 ratio than in the other cases. In vivo recombined vectors were amplified in *E. coli* and sequenced, confirming the successful assembly in all cases: in all, these data suggest that exogenously provided fragments could be assembled in vivo and that the fragment relative abundance plays a marginal role in limiting genomic off-target integration.

**Table 2:** Summary of results for in vivo recombination (3-fragments assembly)

Fragments	Molar ratio	Overlapping ends (nts)	Fluorescent colonies^a^	Coefficient of variation	Fluorescent colonies (%)^b^
1 + 2 + 3	1:1:1	50	47	2.13	65.3
1 + 2 + 3	1:1:1	20	42.5	3.53	59
1 + 2 + 3	1:1:3	50	62.5	4	86.8
1 + 2 + 3	1:1:3	20	63	4.76	87.5
1 + 2 + 3	1:1:6	50	65.5	2.29	91
1 + 2 + 3	1:1:6	20	65	3.07	90.3

^a^Average of colony numbers obtained from 3 independent transformations

^b^Clones are defined as positive if their normalized fluorescent resulted >80 % of the difference between positive and negative controls

## Conclusions

In this technical report, a 452-nt sequence (panARS) was tested for its capacity to confer stable replicative maintenance to a commercial plasmid used for recombinant protein production in *P. pastoris*. Plasmids carrying the panARS sequence complement the existing toolbox for RPP in *P. pastoris*. It is hereby showed that episomal panARS plasmids outperformed the corresponding integrative plasmids in terms of protein production, efficiency of transformation, and macroscopic clonal homogeneity. Endogenous *P. pastoris* sequences were not nearly as efficient in BFP expression compared to panARS-based constructs. At the single cell level, discrete subpopulations of high- and low-producers were consistently detected in clones carrying episomal panARS vectors. In addition, panARS episomal plasmids could be successfully used for quick in vivo self-ligation cloning directly using fragments with overlapping DNA sequences. This method was validated for gene assembly with panARS-based vectors of varying overlapping lengths and molar ratios between fragments: the resulting minimal amount of background, and reported high reproducibility of clone behaviour will allow the application of panARS plasmids to in vivo recombination for synthetic biology applications, where complex, multigene cassettes might be more easily assembled in *P. pastoris*. Taken together, this report characterizes for the first time the use of panARS episomal plasmids to improve the *P. pastoris* molecular toolbox for synthetic biology.

## Methods

### Strains, plasmids, and materials

*Pichia pastoris* strain GS115 (Life Technologies, USA) and *E. coli* Top10F (Life Technologies, USA) were used in this study. Plasmid pSEC-SUMO (Lifesensors, USA) was used as a backbone vector to generate pARS vector for *P. pastoris*. Vector Gateway^®^ TagBFP-AS-C (Evrogen, Russia) was used as the source of a Blue Fluorescent Protein (BFP) gene codon-optimized for *S. cerevisiae*. All primers and gBlocks, available as supplementary information, were synthesized by IDT (Singapore). All restriction enzymes, as well as Gibson cloning kits, were purchased from NEB (Singapore). DNA amplification was performed using Q5 DNA polymerase from NEB (Singapore), while colony PCR for screening was performed with Dream Taq polymerase (Promega, USA) following standard molecular biology protocols [32]. DNA sequencing was performed by Axil Scientific Support (Singapore). All chemicals were purchased from Sigma (USA).

### Cloning and transformation

The BFP gene was amplified from the Gateway^®^ TagBFP-AS-C vector using primers AA_B1_BstB1_BFP_fw and AA_B3_EcoR1_BFP_rev (Table 3). Plasmid pSEC-SUMO was amplified using Q5 polymerase and primers AB_A8_AOX1end_Rv and AB_A9_pARS1_GOIend_Fw, removing the α-mating factor and SUMO coding sequences, and BFP was joined to the backbone vector via Gibson cloning to generate the vector pSEC-BFP. A 452-nt fragment, corresponding to the *pan*ARS sequence [16], was synthesized as a gBlock (IDT, Singapore), and cloned in various positions on the pSEC-BFP vector via Gibson cloning, generating vectors pARS-AOX1-BFP, pAOX1-BFP-ARS, and pARS-oppBFP. All vectors were fully sequenced for confirmation. Approximately 50 ng of the various vectors were used to transform *P. pastoris* GS115, using the condensed electroporation protocol [12]. For transformation with integrative vectors, plasmids were digested at 30 °C for 2 h with *Swa*I, and column purified with Wizard SV Gel and PCR Clean-up System (Promega, USA) prior to transformation. All transformations for in vivo recombination evaluation were performed in duplicate.

**Table 3:** Primers used in this study

Primer name	Primer sequence	Notes
AA_B1_BstB1_BFP_fw	AAGTTCGAAACGATGTCTGAATTGATTAAAGAGAATATG	
AA_B3_EcoR1_BFP_rev	AGGGAATTCTCATTAGTTCAATTTGTGTCCTAACTTA	
AB_A8_AOX1end_Rv	CGTTTCGAATAATTAGTTGTTTTTTGATCTTCT	
AB_A9_pARS1_GOIend_Fw	GAATTCCAACCTGCGATTGATCT	
qPCR-PpACT1-Fw (ACT1, Set 1)	CCATCCATTGTGCACCTCAAG	[35]
qPCR-PpACT1-Rv (ACT1, Set 1)	CGTCTAGAAGCTGAACGACAAG	[35]
qPCR-PpACT1_2_Fw (ACT1, Set 2)	CGAATCTGGACCATCCATTGTG	
qPCR-PpACT1_2_Rv (ACT1, Set 2)	ACGACAAGTAGACACCACCAATC	
qPCR_pEM7_1_Fv (Zeo, Set 1)	ACGACAAGGTGAGGAACTAAACC	
qPCR_ZEO_1_Rv (Zeo, Set 1)	AAGTCGTCCTCCACGAAGTC	
qPCR_pEM7_2_Fv (Zeo, Set 2)	CGACAAGGTGAGGAACTAAACC	
qPCR_ZEO_2_Rv (Zeo, Set 2)	GAAGTCGTCCTCCACGAAGTC	
qPCR_BFP_1_Fw (BFP, Set 1)	AGACGGTGGAGTTTTGACTGC	
qPCR_BFP_1_Rv (BFP, Set 1)	AATGTCTCAGTGAATGCCTCCC	
qPCR_BFP_2_Fw (BFP, Set 2)	ACGGTGGAGTTTTGACTGCTAC	
qPCR_BFP_2_Rv (BFP, Set 2)	TGAATGCCTCCCATCCCAATG	

### Deep well plate cultivation and fluorescence measurement

*Pichia pastoris* cultures were grown in 96 Well Masterblock, 2 mL, V-bottom plates (Greiner, Germany), sealed with BREATHseal™ gas-permeable sealer (Greiner, Germany) in a variant of BMD1 media [33], where 1 % d-glucose was substituted with 1 % sorbitol (BMS media), and using 100 mg/L of zeocin as selection marker. A final concentration of 1 % methanol was added to the media to induce protein production. All cultivations were performed at least in duplicate. After 48 h cell suspensions were diluted 1:10 in PBS and evaluated for OD_600_ and fluorescence emission in a Tecan Infinite^®^ 200 PRO series (Tecan, Austria), using Microplate PS, 96 Well, F-Bottom clear plates (Greiner, Germany) or Microplate PS, 96 Well, F-Bottom Black plates (Greiner, Germany), for OD_600_ or fluorescence respectively; the latter was determined using 402 nm and 452 nm as excitation and emission wavelength, respectively, and normalizing all values against OD_600_. Every reading of both OD600 and fluorescence was performed in triplicate (technical replicates).

### Genomic DNA extraction

20 OD of yeast cultures were pelleted and washed in 500 µL distilled H_2_O. Pellet was re-suspended in breaking buffer (2 % (v/v) Triton X-100, 1 % (v/v) SDS, 100 mM NaCl, 10 mM Tris–Cl pH 8.0, 1 mM EDTA pH 8.0). 200 µL of glass beads and 200 µL of phenol/chloroform/isoamylalcohol were added. Samples were vortexed at highest speed for 5 min, supplemented with 200 µL of TE Buffer (10 mM Tris–Cl, 0.1 mM EDTA pH 8.0), vortexed briefly, and centrifuged at 13,000*g* for 5 min at room temperature. Samples were extracted two more times with phenol/chloroform/isoamylalcohol and one final time with chloroform. 1 mL of 100 % freeze-cold ethanol was added to the aqueous phase, mixing by inversion, and samples were incubated at −20 °C for 1 h. Samples were mixed several times by inversion, centrifuged at 13,000*g* for 3 min at 4 °C and supernatant aspirated. Pellet was air-dried; 400 µL of TE were then added to pellets and incubated at 65 °C for 10 min, followed by addition of 30 µL of RNase A and incubation for 1 h at 37 °C. 10 µL of 4 M ammonium acetate was added, samples were mixed by inversion, and quickly spun. 1 mL of 100 % ethanol (−20 °C) was added and mixed by inversion, followed by 13,000*g* centrifugation (3 min) at 4 °C. Pellet was air-dried, resuspended in 50 µL of TE buffer and incubated in 65 °C water bath for 10 min.

### Picogreen DNA quantification

Lambda DNA (Life Technologies, USA) was serially diluted to produce DNA standards at the following concentrations (ng/µL): DNA Standards Set #1—50, 33.33, 16.66, 8.3, 4.16, 2.08, 1.042, 0.5208, 0; DNA Standards Set #2—75, 50, 25, 12.5, 6.25, 3.125, 1.5262, 0. Quant-iT™ PicoGreen^®^ dsDNA reagent (Life Technologies, USA), diluted 200× in 1x Tris–EDTA (10 mM Tris–HCl, 0.1 mM EDTA, pH 8.0) was transferred to wells of a white 96-well plate (Greiner Bio-One, Germany), 195 μL per well (“DNA sample plate”).

Based on previous spectrophotometer readings (NanoDrop 2000c), genomic DNA samples were diluted 5x, 10x, 20x, or 40x, to fall within the range of the DNA standards made. 2 µL of each lambda DNA standard and diluted genomic DNA sample was transferred to wells of the DNA sample plate. Sample plate was scanned on a SpectraMax M5 microplate reader (Molecular Devices, USA) using the preconfigured Quant-It Picogreen protocol in the accompanying SoftMax Pro software, measuring fluorescence signal at 525 nm (excitation wavelength: 490 nm). Sample concentrations were calculated from RFU readings using the linear regression equation derived from the DNA standards. All samples were evaluated in triplicate.

### Digital droplet PCR (ddPCR) copy number determination

Purified genomic DNA was digested with *Kpn*I (NEB, USA) for 1 h at 37 °C. No heat inactivation was performed, following ddPCR manufacturer indications. An Evagreen ddPCR mastermix was assembled, mixing 10 µL of Eva Green 2x master solution (Biorad, Singapore), 2 µL of primer set (100 nM each, Table 3), 1 µL of genomic DNA sample (0.5 ng/µl, as reported in [34]) and mQ H_2_O up to 20 µL. Primers were designed following published indications [35], although different sets of primers were tested for optimal signal. Following droplet generation with a Droplet generator (Biorad, Singapore), samples were transferred to a 96-well plate and sealed. PCR was performed adjusting the ramp rate on a C1000 Touch Deep Well PCR system (Biorad, Singapore) to 2 °C/s, applying the following cycle: 95 °C, 5 min; 40x (95 °C, 30 s; annealing/extension 57–60 °C); 4 °C, 5 min; 90 °C, 5 min; 4 °C, infinite hold). Droplet detection was carried out in a QX200 Droplet Digital PCR system (Biorad, Singapore) and analysed using the software QuantaSoft v. 1.7.4.0917 (Biorad), following an absolute quantification protocol.

### Copy number determination of in vivo recombined fragments

qPCR was conducted to accurately evaluate the copy number of individual fragments upon in vivo recombination. All qPCR reactions were performed in triplicates for each transformed clone and each diluted standard, using FastStart Essential DNA Green Master (Roche, USA) and LightCycler^®^ 96 Instrument (Roche, USA). A 5 ng DNA sample of each transformed clone was used as template for qPCR reaction. Two primer sets, namely Zeo, Set 1 and BFP, Set 2, were designed to determine the copy number of the two transformed fragments. To determine PCR efficiency of the two primer sets, a 5-log dilution series of PARS-BFP plasmid, started from 10 ng, was used. Each diluted standard was mixed with 5 ng of untransformed parental genomic DNA, to compensate for any non-specific amplification arising from genomic DNA contamination in the plasmid preparation. During the qPCR reaction, the enzyme was activated at 95 °C for 10 min, followed by 45 cycles of 95 °C for 10 s, 57 °C for 10 s and 72 °C for 10 s.

Standard curve of each primer set amplified the 5-log diluted plasmid was plotted, and primer amplification efficiency was determined. The adjusted copy number ratio of the two transformed fragments in each transformed clone was calculated using formula: E_Zeo_^Ct ZEO^/E_BFP_^Ct BFP^, where E_Zeo_ and E_BFP_ are primer efficiencies of Zeo and BFP primer set, respectively, while CtZEO and CtBFP are Ct values of a transformed clone amplified using ZEO and BPF primer set, respectively.

### FACS analysis and hierarchial clustering

Samples were normalized to OD600 = 0.1 using a Tecan EVO 150 liquid-handling robot. After washing with PBS twice, samples were analysed in 96-well batches on a MACSQuant VYB instrument (Miltenyi), acquiring ~80,000 cells per clone. The Blue Florescence Protein signal profile was gated into 10 equally spaced areas (P1–P10) using FlowJo_vX.0.7 software. The blue fluorescence intensity for each gate was visualized in heat maps built using customized R scripts. Hierarchial clustering was generated based on the relative frequencies of fluorescence events in every gate, using MATLAB R2014a (version 8.3.0.532, Mathworks, Natick, MA). An agglomerative ‘bottom-up’ clustering algorithm [36] was used where the fluorescence of each clone initiated as its own cluster, which then merged with clones possessing similar profiles.

### Statistical analysis

Data analysis was performed with GraphPad Prism 6 (GraphPad Software, USA).
